# The essential oil of Brazilian pepper, *Schinus terebinthifolia *Raddi in larval control of *Stegomyia aegypti *(Linnaeus, 1762)

**DOI:** 10.1186/1756-3305-3-79

**Published:** 2010-08-27

**Authors:** Ary G Silva, Drielle L Almeida, Silas N Ronchi, Amarildo C Bento, Rodrigo Scherer, Alessandro C Ramos, Zilma MA Cruz

**Affiliations:** 1 Centro Universitário Vila Velha - UVV. Rua Comissário José Dantas de Melo, 21, Boa Vista, Vila Velha, ES, CEP 29.102-770, Brazil; 2 Tommasi Laboratório. Rua Araribóia, 322, Centro, Vila Velha, ES, CEP.: 29100-340, Brazil; 3 Tommasi Analítica. Avenida Luciano das Neves, 2016, Divino Espírito Santo, Vila Velha, ES, CEP. 29.107-010, Brazil

## Abstract

**Background:**

The ability of mosquitoes of the genus *Aedes *and its allies, such as *Stegomyia*, to transmit diseases such as dengue and yellow fever, makes them important in public health. This study aims to evaluate the use of the essential oil of Brazilian pepper in biological control of by assessing and quantifying the larvicidal effect against *S. aegypti*, the only available access to dengue control, and test its risk of genotoxicity with *Salmonella typhimurium *as an indicator of safety for its environmental use.

**Results:**

The density of the oil was 0.8622 g mL^-1^. Gas chromatography coupled with mass spectrometry revealed six major constituents: δ-3-carene (55.43%), α-pinene (16.25%), sylvestrene (10.67%), germacrene D (2.17), β-myrcene (1.99%), and isoterpinolene (1.4%). The minimum inhibitory dose to larvae development was 862.20 μg mL^-1^. The median lethal dose (LD_50_) of the essential oil for larvae was between the concentrations of 172.44-344.88 μg mL^-1^. There was no mutagenic risk for the essential oil, since there were no biochemical or morphological changes in *S. typhimurium *after exposure to the essential oil.

**Conclusions:**

The minimum inhibitory essential oil concentration and the median lethal dose pointed to the value of the use of water dispersions of Brazilian pepper essential oil as an environmental safe natural larvicidal for *S. aegypti*.

## Introduction

Dengue was eradicated in Brazil in 1955, but outbreaks of this tropical disease have been occurring since the 70 s [1]. In fact, dengue is an enormous local as well as a global problem, because in a hundred million households all over the world, *S. aegypti *is perfectly adapted. This makes the commensal virus that this insect harbors also perfectly adapted to its domesticity, exposing some 2.5 billion people in tropical and subtropical countries to a serial infection of at least one of the four serotypes that have evolved [2].

Because of the growing worldwide burden of dengue fever, it is expected that there will be a corresponding impact on the health sector as well as on the global economy. Total mean costs in International Dollars were estimated in I$514 and I$1,394, for each ambulatory and each hospitalized case, respectively, in five American countries (Brazil, El Salvador, Guatemala, Panama, and Venezuela) and three Asian countries (Cambodia, Malaysia, and Thailand). With an annual average of 574,000 cases reported, the aggregate annual economic cost of dengue for the eight study countries is at least I$587 million. With an adjustment for under-reporting could raise this total to $1.8 billion, and incorporating costs of dengue surveillance and vector control would raise the amount further [3].

One of the main reasons for the rise of epidemics, such as dengue, is the high human population density and unplanned urbanization [4], allied with the high level of domesticity of its vector [2]. The mosquito that acts as the dengue fever vector is *S. aegypti *(Linnaeus, 1762), a new combination for *Aedes aegypti *[5], and it has morphological characteristics that make its identification easy, such as brown wings and a silver-white strip on each side of its thorax [6,7]. They are long distance flyers and are attracted by hosts and by the interaction of physical, chemical, biological and sensorial factors, such as vision and olfaction. Their control can be accomplished in several ways, through chemical, physical, biological and integrated methods [6,8].

The lack of urban planning, sanitation, surveillance, education, and information are intrinsic factors to the challenges of this disease and its vector control [9]. Besides these facts, a major problem for this disease is the lack of vaccines and drugs, which development is complicated by the fact that dengue virus exists as four distinct serotypes. Therefore, a broad robust immune response must be induced simultaneously against all four serotypes while avoiding exacerbating the potential risk of developing the dengue diseases through incomplete immune responses [10]. Therefore, disease control comprises five major components: selective integrated vector control, with community and intersectoral participation; active disease surveillance based on a strong health-information system; emergency preparedness; capacity building and training; and vector-control research [11], especially blocking its reproduction that can occur in any receptacle that accumulates water [12].

Considering the damage caused by diseases such as dengue in tropical and subtropical countries [2], and the biodiversity of plant species in the tropics, studies on the use of plant natural products that demonstrate potential larvicidal effect have increased [13]. Because natural products are usually considered not to disturb the natural ecological balance, besides being naturally available and economically viable [14], and, in nature, they correspond to phytochemicals that were predominantly secondary compounds produced by plants and that protects themselves against herbivorous insects [13].

All over the world, phytochemicals of citronella oil have been used as insect repellents since 1901 [15], and since 1947, there have been several reports of a large number of botanical insecticides containing a wide spectrum of bioactive components, including insecticides [16]. Research attention was focused on the use of South American plants in larvae control of *S. aegypti *at the end of 20th century, concerning plant crude extracts, but without indication of any phytochemicals involved in that effect [17].

Considering Brazilian plant species diversity, this trend to make wide screenings of larvicidal plant extracts is still followed [18,19] and some authors have demonstrated biological activity against the larvae of *S. aegypti*. Using the oil, the essential oil, and ethanolic extracts, also with no elucidation of their phytochemical constitution, 17 plants have shown larvicidal activity [20]. The phytochemical nature of some of the Brazilian natural products has been pointing to terpenes, mainly mono- and sesquiterpenoids, and phenylpropanoids as the active larvicidal constituents [21]. The essential oils achieved more importance in larvicidal screening of plant derivatives and extracts studied in Brazil, even when the plants studied were exotic [22], but the native sources of essential oil were still the investigative focus [23-27], mainly because of the interest in searching for environmentally safe insecticides [27].

*Schinus terebenthifolia *Raddi (Anacardiaceae), commonly known as Brazilian pepper, is an evergreen tree, native of South America, especially Brazil, Paraguay and Argentina. The fruits are drupes and have green color when they are immature, and become dark pink or red, in maturity [28], with one dark brown seed per fruit [29]. The essential oil of the vegetative parts showed non-steroidal anti-inflammatory activity by inhibiting phospholipase A2 [30], acting by competitive inhibition of this specific enzyme [31] due to one of its components, schinolmasticadienoic acid [32]. Its healing activity was also directly related to triterpenoids present in fruits [33]. The essential oil also showed antimicrobial activity by several substances, such as terebinthone, hydroxymasticadienoic, terebinthifolic, and ursolic acids [34], and antifungal activity was also evidenced [35].

Since there is no information about the use of the essential oil of *S. therebintifolia *in larval control of *S. aegypti*, this study aims to investigate biological control of this mosquito by the use of essential oil of Brazilian pepper, as well as to assess and quantify the larvicidal effect, and to evaluate its risk of genotoxicity in *Salmonella typhimurium *ATCC 14028, as an indicator of safety for environmental use.

## Methods

### Essential oil extraction; purification, and density determination

Fruits of the Brazilian pepper tree were collected from specimens occurring in the region of Vitória, Espírito Santo, Brazil, and 250.0 g of those fruits were separated from any impurities in the Laboratory of Plant Ecology, at the Centro Universitário Vila Velha - UVV.

The extraction of essential oil was made by hydrodistillation in a Clevenger apparatus with the crunched fruits and seeds, during one hour of the extraction process. This procedure was performed in the laboratory of Chemical Sciences, at UVV.

After extraction, the essential oil was transferred to a glass vial, and its purification was made by separation of the remnant water by freezing, and the essential oil which was kept in liquid phase, was drained from the vial.

The density of the essential oil was gravimetrically determined by weighing 1 mL of liquid at 20°C, using a temperature-controlled water bath. The essential oil was weighed in an analytical balance with accuracy of 1.0 mg.

### Chromatographic Analysis

The identification of the essential oil components was carried out by high resolution gas chromatography analysis, in the Fine Chemistry Laboratory, at Tommasi Analítica. The injection volume was 2 μL, composed of 1.6 mL of a solution of essential oil (30 mg/ml) and 0.4 mL of a solution of hydrocarbon series of C7-C30, as internal standard, both in *n*-hexane as solvent.

The gas chromatography coupled with mass spectrometry - GC-MS - system used consisted of a gas chromatograph, Thermo Scientific^® ^Ultra GC coupled to a mass spectrometer, Thermo Scientific^®^. The fused silica capillary column used was a DB-5 J & W Scientific (30 m × 0.25 mm × 0.25 mm). Helium was the carrier gas and the column temperature program was increased by 3°C per minute between 60°-240°C. The mass spectra were obtained at 70 eV at a scan rate of 0.84 scan/sec, at the range m/z 40-500 [36].

The retention times of sample components and a mixture of *n*-alkanes from C7-C30, co-injected into the GC-MS system under the same temperature program were used for the calculation of the Kovats Retention Index - KI [36]. Identification of essential oil components was based on the calculated KI compared with the available literature [36], and mass spectra with the GC-MS spectral library.

### Biological assay with larvae of *Stegomyia aegypti*

Larvae of *S. aegypti *in the third instar were obtained after incubation of eggs in the Laboratory of Biomarkers of Environmental Contamination and Genotoxicity, at UVV, using 2,000 mL of distilled water with natural food for fish, composed of dried cysts and eggs of planktonic organisms. The eggs of *S. aegypti *were supplied by the Center for Zoonosis Control from the Health Department at Vitoria, Espírito Santo state, Brazil.

A series of tubes was prepared in five replicates for each control, blank and treatment performed, and each replicate received 10 larvae, including a control with distilled water, and a blank with an aqueous solution of 0.5% Tween 80 that was used as dispersing medium for the essential oil and exposure of larvae to treatment.

Since the lethal dose of the essential oil was 862.20 μg mL^-1 ^[37], a concentration gradient was prepared, ranging from 86.22 up to 862.20 μg mL^-1^, by the oil dispersion in a solution of Tween 80 at the concentration of 0.5%. Afterwards, 10 dilutions were prepared at growing rate of 86.22 μg mL^-1^, with the ability of larval locomotion for respiration as the survival criterion. Assessments of the surviving larvae were made 24, 48 and 72 hours after inoculation, to safely avoid the emergence of adult mosquitoes.

### Statistical data analyses

The percentages of larval death for each replication were transformed by the arc-sine square root of its proportions, and its normality was verified by a K^2 ^test, based on the deviations from symmetry and kurtosis of the probability distribution curve of the data obtained in relation to the null hypothesis of a normal distribution. Another assumption for parametric tests, homogeneity of variance was verified by the Bartlett test [38].

Since mortality rates could not be normalized by arc-sine transformation, an ordinal logistic regression was performed, taking the dose and duration of exposure to the essential oil as independent variables and larval mortality rates as the dependent variable. The null hypothesis tested was that the larval mortality rate was independent of dose or time of exposure to essential oil. Odds ratios were estimated for each analyzed parameter [38]. The LC_50 _was calculated from the equation of straight line generated by the linear model tested [39].

### Genotoxicity testing

*S. typhimurium *ATCC 14028 was exposed to essential oil at the concentration of 862.20 mg mL^-1 ^dispersed in Bovine Brain Heart Infusion Broth - BHI. Bioassays were made in triplicates using only BHI broth as the control, and a blank assay containing the broth medium and Tween 80 at 0.5%, as surfactant, in the Laboratory of Environmental Microbiology and Biotechnology, at UVV.

Inocula were made by suspending the microorganism in water, at standard 1 in Macfarland's scale, from which 100 μL were inoculated in BHI broth, and then incubated at 37°C for 24 hours. Afterwards, 10 μL of this broth were inoculated in triplicates on BHI solid medium, for the control, blank and exposed microorganisms, and then incubated at 37°C for 24 hours.

Bacteria isolated from those treatments were evaluated concerning their colonial morphology and their biochemical capacity of acid production by the use of lysine, ornithine, and arginine. All inocula for biochemical testing were prepared from the same 18-24 hours subculture from a BHI agar, in a MicroScan WalkAway 968^®^, in Laboratory of Microbiology, at Tommasi Laboratório.

## Results and discussion

### Essential oil extraction purification and density determination

After purification, 10.25 mL of essential oil were obtained, with a density of 0.8622 g mL^-1^, representing 3.54% yield of fresh fruits. This result was different from that found by Cole [40] who obtained a specific gravity of 0.9097 ± 0.02 g.mL^-1^. This was probably because of changes in the quantity and quality of the oil constituents caused by different environmental factors related to soil, sun exposure, amount of water and other external factors.

### Chromatographic Analysis

**Figure 1:**
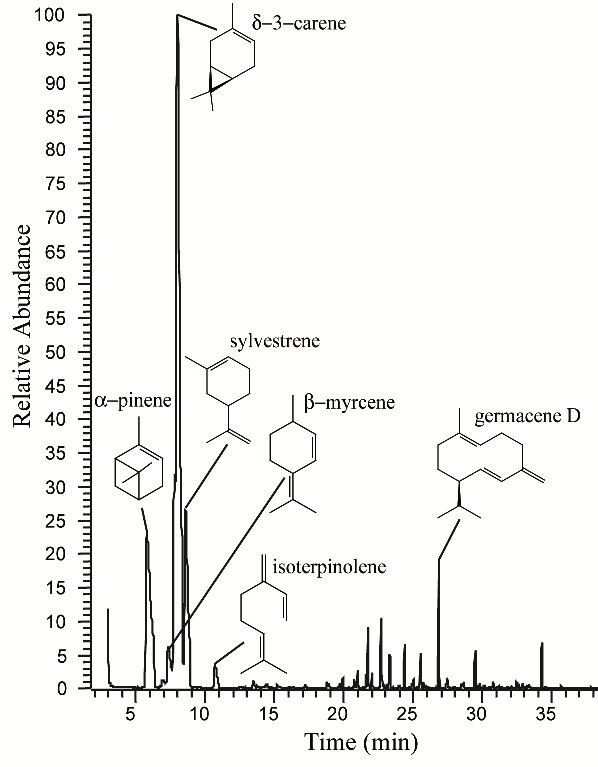
**Chromatographic profile and chemical structure of major constituents of the essential oil from fruits and seeds of *Schinus terebinthifolia***.

The separation of the essential oil components revealed that the chromatographic profile (Figure 1) was predominantly composed of terpenic compounds (Table 1) in total 86.51%, and having as the main constituents δ-3-carene (55.43%), the α-pinene (16.25%), the sylvestrene (10.67%). Among the minor components are sesquiterpenic compounds (2.17%), such as the β-myrcene (1.99%).

**Table 1:** Major constituents of essential oil from fruits of *Schinus terebinthifolia *Raddi, determined by gas chromatography coupled to mass spectrometry.

Kovats indices		Substance	(%)
Calculated	**Adams **[36]		
1013	1011	δ-3-carene	55.43
936	939	α-pinene	16.25
1029	1027	sylvestrene	10.67
1477	1480	germacrene D	2.17
991	991	β-mircene	1.99
1087	1086	isoterpinolene	1.40

One of first studies with Brazilian pepper essential oil reported that its main constituents were α-pinene (12.94%), β-pinene (5.02%), α-phellandrene (13.04%), δ-3-carene (29.22%) and β-phellandrene (18.08%) [41]. Another study shows that the major components of the oil were δ-3-carene (30.37%), limonene (17.44%), α-phellandrene (12.60%), α-pinene (12.59%), myrcene (5.82%), and *o*-cymene (3.46%) [40]. In other study the main constituents were sabinene, α-pinene, caryophyllene and germacrene D [42].

These qualitative and quantitative variations in this essential oil components may be related to the environmental conditions to which the plant is exposed such as mineral and water supply and sunlight [40].

### Bioassay with larvae of *Stegomyia aegypti*

The minimum inhibitory dose to larval mortality was 862.20 μg mL^-1 ^and near 50% of the larvae death occurred between the essential oil concentrations of 172.44-344.88 μg mL^-1^. There was no larval mortality in the control, even after 72 hours (Table 2). Other studies have shown that the LD_50 _of the essential oil of *S. terebinthifolia *Raddi was 117.34 μg mL^-1 ^[40], which did not correspond to the value found in this study. This fact may be closely related to the oil density, as a reflex of the qualitative and quantitative change in the concentrations of their constituents that may influence the calculation of median lethal dose [36,40].

**Table 2:** Larval mortality of *Stegomyia aegypti *(Linnaeus, 1762) in a concentration gradient of the essential oil from fruits and seeds of *Schinus terebinthifolia *Raddi, the Brazilian pepper, in a 72 hour long bioassay.

essential oil gradient concentration (μg mL^-1^)	Larval mortality (% Mean ± SE)		
	24 h	48 h	72 h
Control	0.00 ± 0.00	0.00 ± 0.00	0.00 ± 0.00
Blank	0.00 ± 0.00	4.00 ± 4.00	4.00 ± 4.00
86.22	0.00 ± 0.00	2.00 ± 2.00	4.00 ± 2.45
172.44	42.00 ± 22.00	48.00 ± 19.85	52.00 ± 18.00
258.66	26.00 ± 19.39	32.00 ± 17.45	32.00 ± 22.00
344.88	24.00 ± 12.08	38.00 ± 14.97	42.00 ± 18.85
431.10	74.00 ± 14.00	82.00 ± 9.17	88.00 ± 8.00
517.32	60.00 ± 24.49	64.00 ± 22.27	64.00 ± 22.27
603.54	60.00 ± 24.49	62.00 ± 23.32	64.00 ± 22.27
689.76	62.00 ± 23.32	64.00 ± 22.27	68.00 ± 19.85
775.98	80.00 ± 20.00	80.00 ± 20.00	82.00 ± 18.00
862.20	100.00 ± 0.00	100.00 ± 0.00	100.00 ± 0.00

SE: Standard Error

Binary logistic regression (Table 3) demonstrated that larval mortality of *S. aegypti*, in spite of being high significantly affected by the concentration of the essential oil and by the incubation time, the interaction between them was not significant. The model that contained the essential oil concentration and the incubation time, without the interaction between them, as well as the models using those two variables independently were highly significant. However, the odds ratio values near 1.00 indicates a low prediction power for mortality for both of them, although the incubation time had showed a better fit to experimental data (Hosmer χ^2 ^= 0.004, *p *= 0.95).

**Table 3:** Binary logistic regression of larval mortality of *Stegomyia aegypti *(Linnaeus, 1762), considering the influence of concentration of Brazilian pepper, *Schinus terebinthifolia *Raddi, essential oil and the incubation time.

Model	Coefficient	Standard Error	*Z*	*p*	Odds Ratio and limits (95%)		
					Ratio	Inferior	Superior
Interaction of essential oil concentration and Incubation time							
Constant	-2.889	0.334	-8.65	0.00**			
Concentration	0.006	0.001	8.70	0.00**	1.01	1.00	1.01
Time	0.020	0.006	3.22	0.00**	1.02	1.01	1.03
Concentration × Time	0.000	0.000	-1.87	0.06^ns^			
G = 514.5; *p *< 0.01. df = 3; Hosmer χ^2 ^= 82.65. *p *< 0.01**							

Essential oil concentration + incubation time							
Constant	-2.392	0.193	-12.42	0.00**			
Concentration	0.005	0.000	19.20	0.00**	1.00	1.00	1.01
Time	0.010	0.003	3.26	0.00**	1.01	1.00	1.02
G = 510.96. *p *< 0.01. df = 2; Hosmer χ^2 ^= 57.26; *p *< 0.01**							

Essential oil concentration							
Constant	-1.911	0.118	-16.20	0.00**			
Concentration	0.005	0.000	19.20	0.00**	1.00	1.00	1.01
G = 500.25. *p *< 0.01. df = 1; Hosmer χ^2 ^= 87.03. *p *< 0.01**							

Incubation time							
Constante	-0.246	0.131	-1.88	0,06^ns^			
Time	0.007	0.003	2.77	0,01**	1.00	1.00	1.01
G = 7.72. *p *< 0.01. df = 1; Hosmer χ^2 ^= 0.004. *p *= 0.95^ns^							

Z: standard coefficient; *p*: significance level; **highly significant; df: degrees of freedom.

This result shows that the larvae are susceptible to the essential oil composition, and the use of natural products can be considered an important alternative for the control of the mosquitoes, since they are biodegradable and do not harm the environment.

### Genotoxicity test

After exposure of the microorganism to the essential oil, we observed no differences in colony morphology in sub-cultures obtained from control, test, and blank tubes. The colonies remained smooth, opalescent and light brightness colonies.

There was also no change in the pattern of the biochemical profile of *S. typhimurium*, for both microorganisms from the control and blank sub-cultures, and those ones obtained from exposure to the essential oil. They preserved their metabolism, typically positive for lysine and ornithine, and negative for arginine.

## Conclusion

The density presented by the oil on which this study was based was 0.8622 g.mL^-1 ^which does not correspond to the density values found in other references, a fact explained by the oil composition differs with regard to quantity concentration of major constituents of the oil.

Gas chromatography coupled with mass spectroscopy (GC-MS) revealed six major constituents: δ-3-carene (55.43%), α-pinene (16.25%), sylvestrene (10.67%), germacrene D (2.17), β-myrcene (1.99%), and isoterpinolene (1.4%). Those major compounds, when added together represent 86.51% of the essential oil of *S. terebinthifolia*.

The larval median lethal dose (LD_50_) of the essential oil was between the concentrations of 172.44-344.88 μg mL^-1^; there was no larvae mortality in the control, even after 72 hours. This value is not in accordance with previous studies, possibly because of the density value, constituents and concentrations of constituents in the oil.

There was no effect of the essential oil from the fruits of *S. terebinthifolia *on aspects of the biochemistry or morphological changes in the experiment with *S. typhimurium*.

## Competing interests

The authors declare that they have no competing interests.

## Authors' contributions

AGS carried out the statistical data analyses as well as the extraction, purification, and chemical analysis of essential oil, microbiological and larvae bioassays. DLA and SNR carried out the extraction, purification, and chemical analysis of essential oil, microbiological and larval bioassays; ACM and ACR carried out the microbiological analysis, RS carried out the gas chromatography analysis, ZMAC carried out the larval bioassays. All authors read and approved this final manuscript.
